# Orthodontic Treatment of Ankylosed Maxillary Incisor through Osteogenic Distraction and Simplified Biomechanics

**DOI:** 10.1155/2019/8152793

**Published:** 2019-07-14

**Authors:** Daniel Gheur Tocolini, Priscila de Oliveira Silva, Iduilton Grabowski, Julia Carelli, Nathaly Dias Morais, Gisele Maria Correr, Francielle Topolski, Alexandre Moro

**Affiliations:** ^1^School of Health and Biological Sciences, Universidade Positivo, Curitiba, Paraná, Brazil; ^2^Department of Orthodontics, Centro de Pós-Graduação Thum/Avantis, Joinville, Santa Catarina, Brazil; ^3^Department of Orthodontics, Federal University of Paraná, Curitiba, Paraná, Brazil

## Abstract

Ankylosed teeth may have a significant esthetic and functional impact especially at the anterior segment of the upper arch. Treatment of ankylosed teeth is challenging. The objective of this case report is to describe a clinical case in which an ankylosed tooth was treated with the use of osteogenic distraction associated with simplified orthodontic biomechanics. A 17-year-old female Caucasian patient presented with a Class II malocclusion, severe maxillary dental crowding, moderate mandibular dental crowding, anterior open bite, upper midline deviation to the right, and upper right central incisor in infraocclusion due to ankylosis. Treatment involved the use of the ankylosed tooth as anchorage for the distalization of the right upper segment to correct the Class II malocclusion and to create space prior to surgery. After one week of surgical osteotomy, traction of the tooth and bone segment was initiated with the use of intermaxillary elastics. The ankylosed tooth was moved to the desired position. Bone formation and mucogingival tissue adaptation were observed. Thus, esthetic and functional improvement was achieved. Osteogenic distraction associated with simplified orthodontic biomechanics is an alternative to the treatment of ankylosed teeth which can replace the use of distractor screws, making treatment simpler and more accessible.

## 1. Introduction

Tooth ankylosis is the fusion of cementum with the alveolar bone. It can be caused by genetic predisposition, local metabolic changes, dental trauma [1], or replantation of avulsed teeth [2]. Ankylosis should be visible radiographically as an interruption in the periodontal membrane space [3]. Clinically, it can be diagnosed by typical metallic sounds upon percussion, lack of tooth mobility [4], dental infraocclusion, and inability of orthodontic movement [3].

Due to the failure of movement with normal vertical dentoalveolar growth, ankylosed teeth and their gingival margin remain infrapositioned relative to adjacent teeth, which is an important esthetic problem, especially in the anterior region of the arch [3, 5]. Treatment of ankylosed teeth, therefore, is challenging. Among the possibilities are surgical luxation or tooth extraction and restoration of the space with prosthetics or implants. Another treatment alternative for ankylosed teeth is osteogenic distraction (OD) [3].

OD is a tissue engineering technique in which the gradual separation of surgically sectioned bone edges results in the generation of a new bone [6]. It is a technique for reconstructing and correcting skeletal deformities, which involves controlled, gradual displacement of a surgically created fracture. As a result, a segment of a mature bone is moved and a new bone is regenerated in the distracted osteotomy site [7, 8].

After osteotomy and placement of a distraction appliance, the bone fragment is kept stable during a latency phase. This allows initial bone healing in the gap between the edges of the fracture and a soft callus is formed [9]. At the end of the latency phase, the distraction is started slowly by separating the bone ends. It is during this phase that new bone formation will be induced by osteogenesis in the spaces around the distracted fragments. When the bone deposited is sufficient, the distraction phase is interrupted and the bone fragment is kept stable, enabling consolidation. At this phase, the bone matures and remodels [10]. As it is a gradual process, OD allows expansion and regeneration of soft tissue associated with the transported skeletal segment [8].

The objective of this case report is to describe a clinical case in which an ankylosed maxillary central incisor was moved to the desired position with OD using simplified orthodontic biomechanics.

## 2. Case Presentation

**Figure 1:**
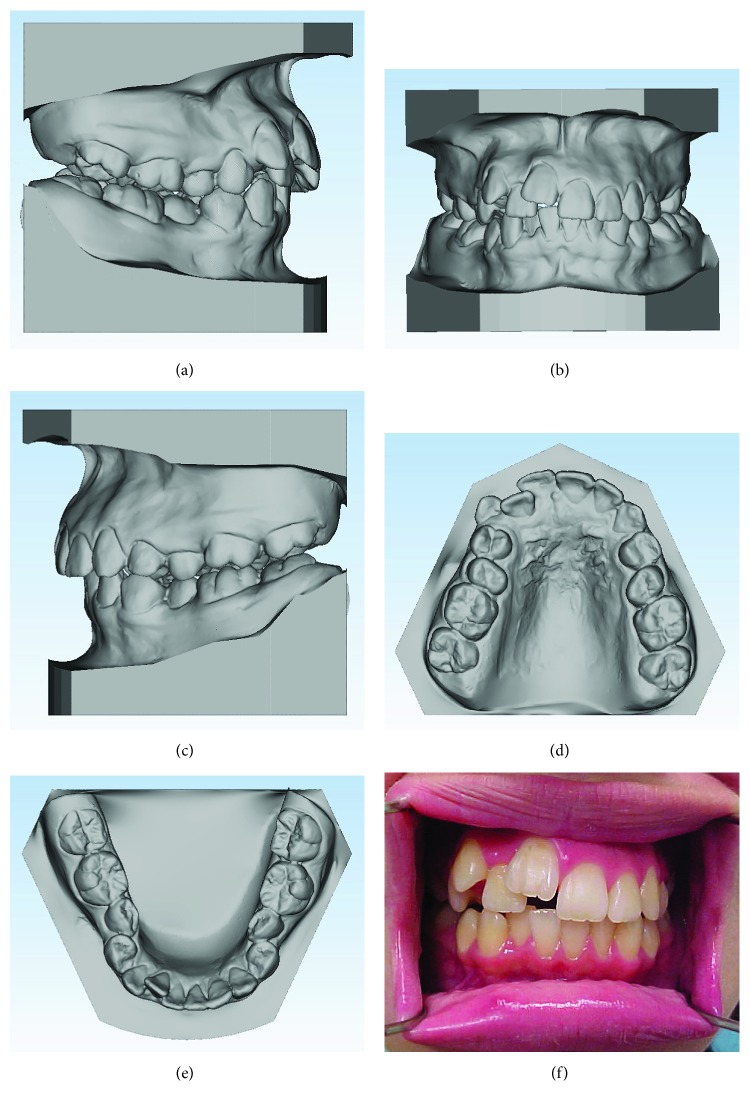
Pretreatment dental casts and frontal intrabuccal view.

**Figure 2:**
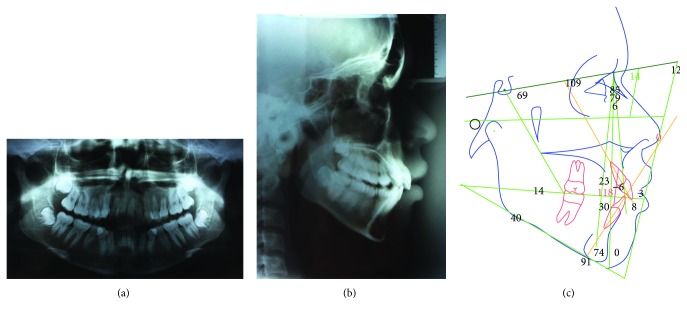
Pretreatment records: panoramic radiograph, lateral ceph and cephalometric tracing.

The patient was a 17-year-old Caucasian female who wanted a more esthetic smile through orthodontic treatment. She had a Class II division 1 malocclusion, severe crowding in the maxillary arch, moderate crowding in the mandibular arch, anterior open bite, upper midline deviation to the right, and the maxillary right central incisor in infraversion (Figure 1). The panoramic radiograph showed the complete development of the teeth, except for the third molars which were still in development. The patient had an asymmetrical face, convex profile, and lack of lip seal (Figure 2). Growth pattern was vertical with a skeletal Class II malocclusion, according to the cephalometric values shown in Table 1. Due to the great esthetic impact of the malocclusion, the patient refused to perform the initial extrabuccal photographs.

The patient and her parents were asked about any history of trauma. They reported that the patient had suffered a fall in childhood, which could have generated ankylosis of the right upper central incisor.

**Figure 3:**
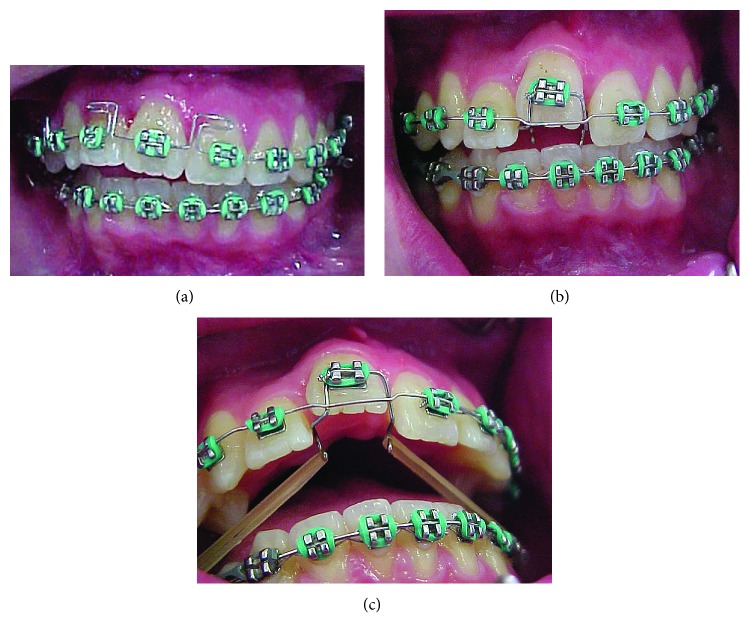
Frontal intrabuccal view after initial alignment and biomechanics used to try to move tooth 11.

Initially, the treatment objectives consisted of alignment and leveling of the dental arches and Class II correction with the use of elastics. Lower premolar-to-premolar slices were planned to create space for dental alignment. However, during the initial alignment and leveling, it was noted that the maxillary right central incisor did not move, which led to a unilateral right open bite. The ankylosis of the maxillary right central incisor was then confirmed. An attempt was made to traction the ankylosed tooth using a .018^″^ segmented wire together with 5/16 medium elastics with 50g of force on each side, 16 hours per day, but it was not successful (Figure 3). The case was then replanned.

Treatment alternatives included extracting the ankylosed tooth and closing the space with orthodontics; extracting the ankylosed tooth, performing a bone graft and inserting a dental implant; and performing osteotomy surgery and OD. The last alternative was chosen.

Treatment plan included the extraction of the four first maxillary and mandibular premolars aiming to correct the Class II malocclusion and especially to open space mesial and distal to the ankylosed tooth in order to perform the osteotomy surgery.

**Figure 4:**
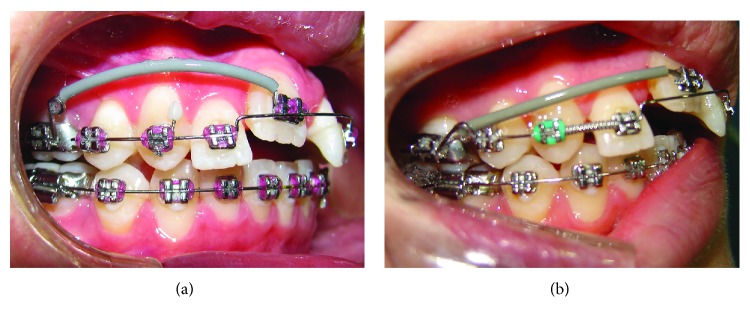
TMA cantilever for Class II correction; reactivation after 2 months and aid of a NiTi open coil spring.

For the correction of Class II relationship, the ankylosed tooth was used as anchor for a distalization cantilever made with titanium molybdenum alloy (TMA) wire (Figure 4).

**Figure 5:**
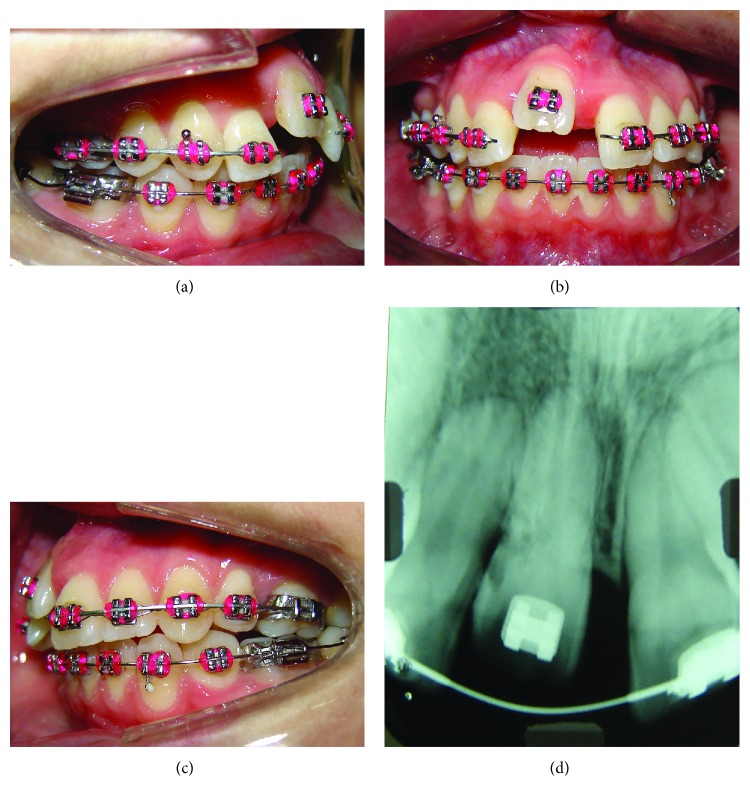
Intrabuccal photographs and periapical radiograph before surgery.

Once the Class II molar relationship was corrected and the spaces were opened, a segmented stainless steel .019^″^ × .025^″^ wire was placed in the upper arch (Figure 5) and a simulation of tooth movement was performed using a plaster model in order to plan the surgery.

The patient was then submitted to surgery. Two vertical vestibular relaxing incisions were performed distal to the upper canines, and a horizontal incision was performed superiorly, in the alveolar mucosa. After the opening of the flap, vestibular periosteum detachment was performed. The mucosa and the periosteum in the palatal region were maintained intact in order to preserve blood irrigation. The osteotomy was performed with drill and chisel mesially, distally, and apically to the root of tooth 11. A chisel was used to release the bone fragment, which remained connected only by the palatal periosteum. After that, the vestibular periosteum and the flap were repositioned and the suture was performed.

**Figure 6:**
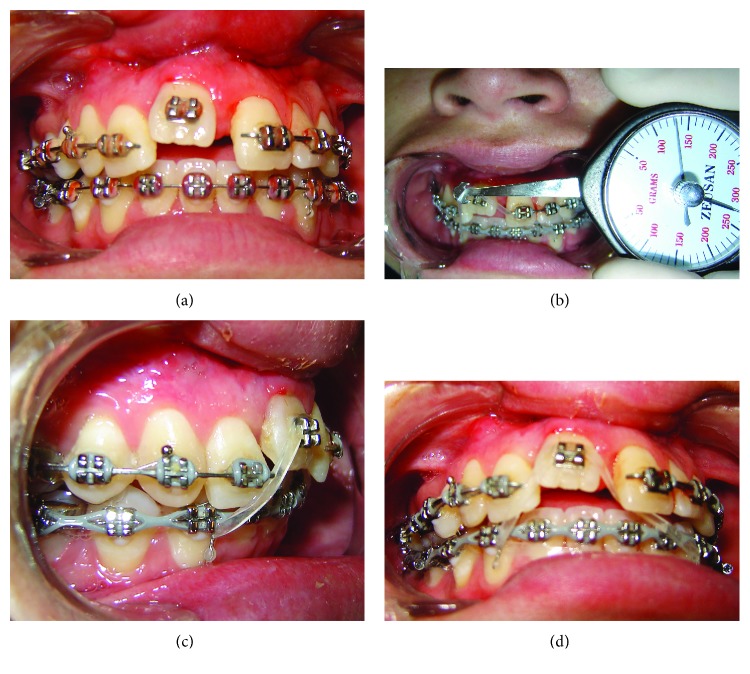
Intrabuccal photographs 1 week after osteotomy; onset of OD with intermaxillary elastics.

**Figure 7:**
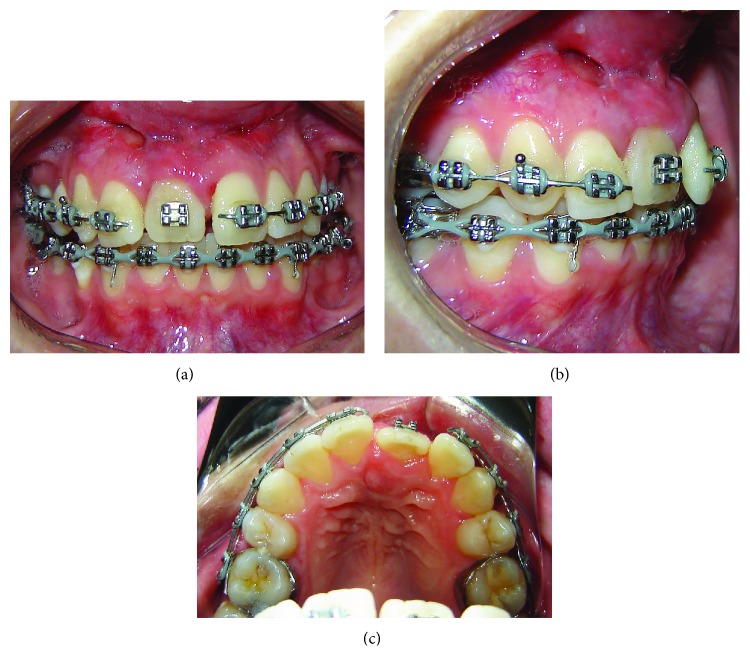
Intrabuccal photographs 15 days after beginning of OD.

After 7 days of latency, OD was initiated using intermaxillary elastics with a force of 320 g (Figure 6). The patient returned 15 days after the onset of OD (Figure 7). When the right upper central incisor reached its correct position, stabilization was started with stainless steel .019^″^ × .025^″^ wire with *in* and *out* bends for 6 months.

**Figure 8:**
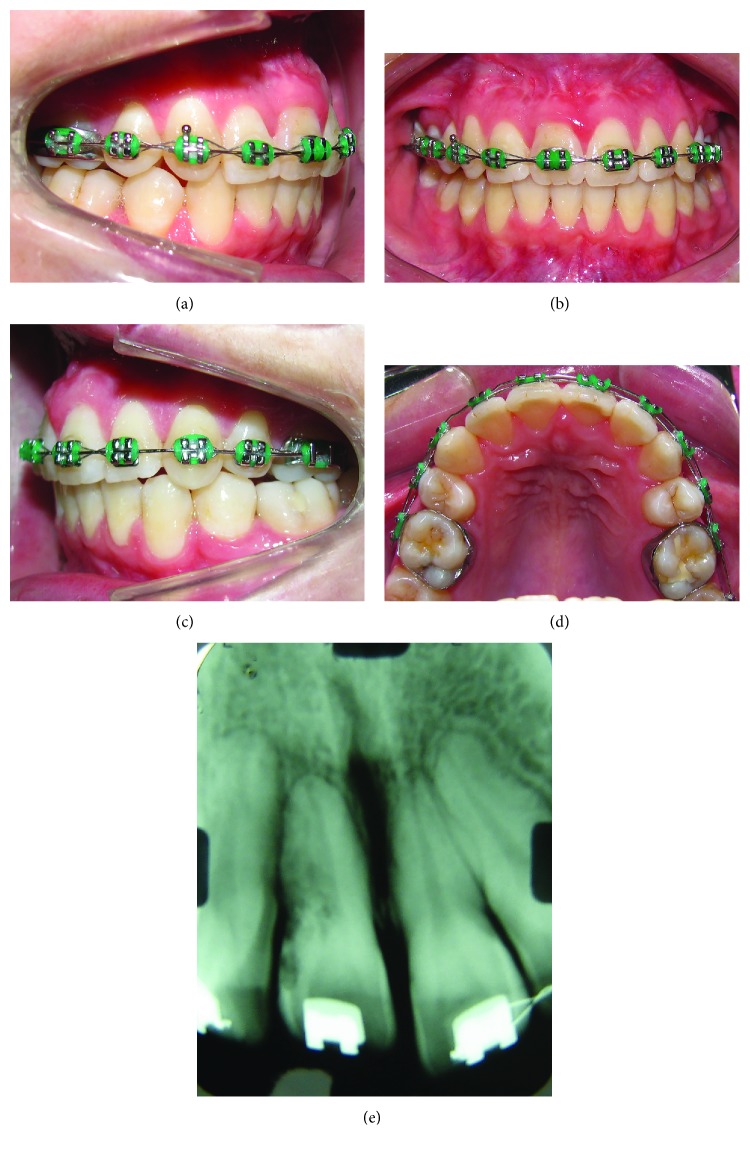
End-of-treatment intrabuccal photographs and periapical radiograph.

Considering the less invasive technique used and taking into account the difficulty in dealing with a case of dental ankylosis in the upper anterior region, the methods used were successful for both the functional and esthetic aspects.  Figure 8 shows the end of treatment intrabuccal photographs and periapical radiograph.

**Figure 9:**
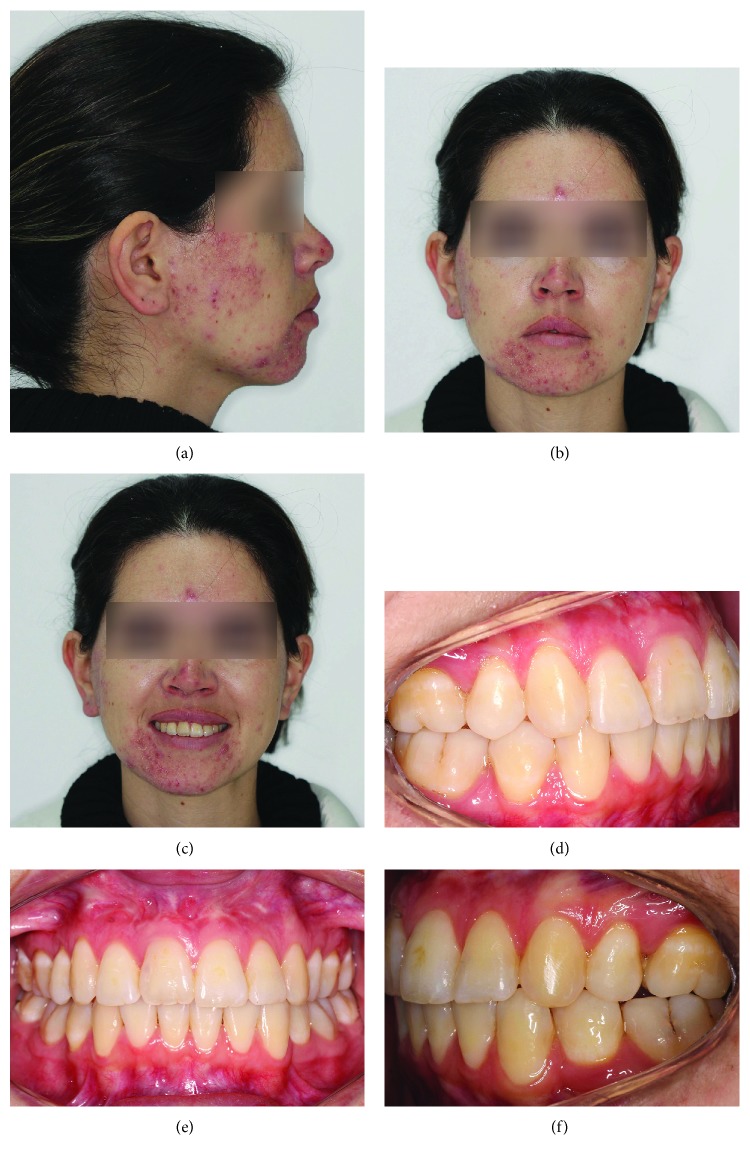
Facial and intrabuccal photographs at 3 years and 4 months after the end of treatment.

**Figure 10:**
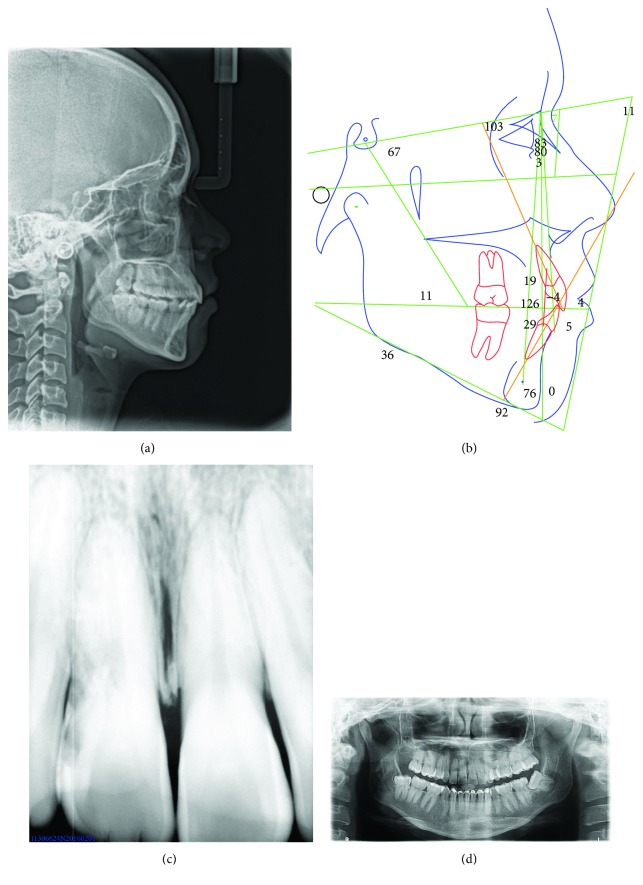
Records at 3 years and 4 months after the end of treatment: lateral ceph, cephalometric tracing, periapical radiograph, and panoramic radiograph.

Figure 9 shows 3 years and 4 months posttreatment intrabuccal and extrabuccal photographs.  Figure 10 shows 3 years and 4 months posttreatment records. Table 1 shows the initial and final cephalometric values.

## 3. Discussion

Infrapositioned teeth may be part of a malocclusion and the possibility of ankylosis must be considered in these cases [5]. However, clinical and radiographic diagnosis of ankylosis is difficult and often this problem will only be confirmed after the start of orthodontic treatment, by the absence of tooth movement [3]. Thus, treatment plans then need to be modified [5].

Treatment of ankylosed teeth is challenging, especially at the anterior segment of the upper arch. One option is the surgical luxation in an attempt to break the fusion between the cementum and the bone. However, a repair process that usually results in recurrence of the ankylosis follows this procedure [3].

Another treatment alternative is tooth extraction and replacement with a prosthetic tooth or an implant. The problem related to this approach is the bone defect that is frequently present due to the failure of vertical alveolar growth associated with the ankylosis [3]. In addition, ankylosed tooth extraction could also result in bone loss, once fusion of cementum to the alveolar bone could lead to the need for bone removal so that tooth could be extracted. Such a vertical alveolar defect could compromise the esthetic outcome of the prosthetic/implant replacement or make the treatment with an implant unfeasible [3]. A bone graft could be necessary, making the treatment even more complex. Orthodontic closure of the space could also be an option. However, the vertical bone defect could impair dental movement or lead to an unpleasant esthetic result.

Other approaches involve corticotomy, osteotomy, and OD [3]. OD is the most efficient technique for positioning ankylosed teeth. It allows the tooth to be moved to the desired position in the arch, along with the supporting tissues. The alveolar process is elongated and its vertical growth deficiency is corrected [5, 7]. Both the incisal edge and the gingival margin of the clinical crown are brought to the proper height in the arch relative to the adjacent teeth [3]. However, although it has advantages, OD also has some drawbacks. The distractors used to move the bone segments are bulky, expensive, and difficult to place in the dental region. Besides that, a second surgery is needed to remove the distractors [5].

Considering this, we opted for OD using simple orthodontic biomechanics with the use of intermaxillary elastics. Dolanmaz et al. [5] also used simplified biomechanics for OD. After osteotomy, the authors performed OD with the fixed appliance arch. Isaacson et al. [3], in a similar clinical case, attempted to reposition the bone fragment and ankylosed tooth at the time of surgery. They did not have success due to the resistance of the soft tissues. They then also used the orthodontic arch to refine the tooth position. An initial bend of 1 mm was built into the arch wire and this procedure was repeated after 2 and 4 weeks.

Kofod et al. [7] argue that this type of biomechanics could not be directly characterized as OD because displacement of the bone segment did not increase gradually. Ilizarov [11] suggested the distraction rate to be 1 mm per day. With this simplified biomechanics, it is not possible to control the amount of separation of the bone fragment per day. However, in the case presented in this article, as in other clinical cases [3, 5], the use of simplified biomechanics was successful in bone and dental repositioning. Dolanmaz et al. [5] emphasize that success may be related to the short distance the tooth should move to its alignment. For longer distances, this type of biomechanics should be considered with caution.

Another aspect that must be considered is the fact that OD using screws allows only unidirectional movements. The use of the arch wire or intermaxillary elastics to distract the bone fragment allows the movement of the tooth and bone in three dimensions, which can lead to a more esthetic result. Kinzinger et al. [12] described a clinical case in which an ankylosed upper central incisor was treated with OD using screws. The tooth was moved to the occlusal plane, but with a marked palatal deviation. Thus, the authors opted to apply forces to the tooth and bone segment using a standard orthodontic appliance before the end of the consolidation phase, in a concept called floating bone effect. For biomechanics with intermaxillary elastics, particular attention should be given to patient collaboration, which is not so critical with the use of screws or the orthodontic arch [12].

The opening of space so that a bone fragment of adequate size can be obtained is fundamental to guarantee the maintenance of the blood supply and the success of the procedure [3, 7, 10, 13]. For this purpose, in the clinical case described, it was decided to extract the four first maxillary and mandibular premolars. Moreover, distalization biomechanics with a .019^″^ × .025^″^ TMA cantilever was used. The ankylosed tooth was used as anchorage for the distalization biomechanics, as suggested by Isaacson et al. [3] and Kofod et al. [7]. Another important point is that osteotomies should be parallel or diverge to the direction the tooth and bone fragment will be moved [3, 7].

Growing patients should be warned about the possibility of relapse, since the OD corrects the dental positioning but does not treat the ankylosis. Thus, the lack of vertical growth of the alveolar bone will persist. In such cases, delaying treatment until growth has ceased must be considered [3]. For patients who have stopped growing, as in the case presented, the possibility of relapse is minimal [7].

In the clinical case presented, the patient did not show signs of loss of pulp vitality of the ankylosed tooth. However, this is a possible complication of this type of treatment [3].

## 4. Conclusion

The treatment resulted in correct positioning of the ankylosed tooth along with the supporting tissues, thus providing better function and smile esthetics. For the clinical case presented, osteogenic distraction with a simplified orthodontic biomechanics was an alternative to using distractor screws, making the treatment simpler and more accessible.

## Figures and Tables

**Table 1:** Cephalometric variables.

Variables	Initial	Posttreatment
Maxillary component		
SNA (°)	85.5	83.8
Mandibular component		
SNB (°)	79.6	80.4
Maxillomandibular sagittal relationship		
ANB (°)	6.4	3.3
Vertical relationship		
FMA (FH-MP) (°)	31.6	29.8
SN-GoMe (°)	40	36.5
*y*-axis (NSGn) (°)	69.7	67.6
Dentoalveolar component		
Mx1.NA (°)	23.7	19.3
Mx1-NA (mm)	3.6	4.1
Md1.NB (°)	30.8	29.9
Md1-NB (mm)	8.2	5.5
IMPA (L1-MP) (mm)	91.7	92.8

## References

[1] Biederman W. (1962). Etiology and treatment of tooth ankylosis. *American Journal of Orthodontics*.

[2] Campbell K. M., Casas M. J., Kenny D. J. (2007). Development of ankylosis in permanent incisors following delayed replantation and severe intrusion. *Dental Traumatology*.

[3] Isaacson R. J., Strauss R. A., Bridges-Poquis A., Peluso A. R., Lindauer S. J. (2001). Moving an ankylosed central incisor using orthodontics, surgery and distraction osteogenesis. *The Angle Orthodontist*.

[4] Lim W. H., Kim H. J., Chun Y. S. (2008). Treatment of ankylosed mandibular first permanent molar. *American Journal of Orthodontics and Dentofacial Orthopedics*.

[5] Dolanmaz D., Karaman A. I., Pampu A. A., Topkara A. (2010). Orthodontic treatment of an ankylosed maxillary central incisor through osteogenic distraction. *The Angle Orthodontist*.

[6] Faber J., Azevedo R. B. d., Báo S. N. (2005). Dentofacial applications of distraction osteogenesis: the state of the art. *Revista Dental Press de Ortodontia e Ortopedia Facial*.

[7] Kofod T., Würtz V., Melsen B. (2005). Treatment of an ankylosed central incisor by single tooth dento-osseous osteotomy and a simple distraction device. *American Journal of Orthodontics and Dentofacial Orthopedics*.

[8] Millwaters M., Sharma P. K. (2015). The role of distraction osteogenesis in patients presenting with dento-facial deformity – an overview. *Seminars in Orthodontics*.

[9] De Bastiani G., Aldegheri R., Renzi-Brivio L., Trivella G. (1987). Limb lengthening by callus distraction (callotasis). *Journal of Pediatric Orthopedics*.

[10] Aronson J., Good B., Stewart C., Harrison B., Harp J. (1990). Preliminary studies of mineralization during distraction osteogenesis. *Clinical Orthopaedics and Related Research*.

[11] Ilizarov G. A. (1989). The tension-stress effect on the genesis and growth of tissues: part II. The influence of the rate and frequency of the distraction. *Clinical Orthopaedics and Related Research*.

[12] Kinzinger G. S. M., Jänicke S., Riediger D., Diedrich P. R. (2003). Orthodontic fine adjustment after vertical callus distraction of an ankylosed incisor using the floating bone concept. *American Journal of Orthodontics and Dentofacial Orthopedics*.

[13] Senışık N. E., Koçer G., Kaya B. Ü. (2014). Ankylosed maxillary incisor with severe root resorption treated with a single-tooth dento-osseous osteotomy, vertical alveolar distraction osteogenesis, and mini-implant anchorage. *American Journal of Orthodontics and Dentofacial Orthopedics*.

